# Centralized Colorectal Cancer Screening Outreach in Federally Qualified Health Centers: A Randomized Clinical Trial

**DOI:** 10.1001/jamanetworkopen.2024.46693

**Published:** 2024-11-25

**Authors:** Daniel S. Reuland, Meghan C. O’Leary, Seth D. Crockett, Deeonna E. Farr, Renée M. Ferrari, Teri L. Malo, Alexis A. Moore, Connor M. Randolph, Shana Ratner, Lindsay R. Stradtman, Christina Stylianou, Kevin Su, Xianming Tan, Van Tang, Stephanie B. Wheeler, Alison T. Brenner

**Affiliations:** 1 Lineberger Comprehensive Cancer Center, The University of North Carolina at Chapel Hill; 2 Department of Medicine, Division of General Medicine and Clinical Epidemiology, University of North Carolina School of Medicine, Chapel Hill; 3 Department of Health Policy and Management, Gillings School of Global Public Health, University of North Carolina School of Medicine, Chapel Hill; 4 Division of Gastroenterology and Hepatology, Oregon Health & Science University, Portland; 5 Department of Health Education and Promotion, College of Health and Human Performance, East Carolina University, Greenville, North Carolina; 6 Department of Maternal and Child Health, Gillings School of Global Public Health, The University of North Carolina at Chapel Hill; 7 Patient Support Pillar, American Cancer Society, Kennesaw, Georgia; 8 Department of Health Behavior, Gillings School of Global Public Health, The University of North Carolina at Chapel Hill; 9 Department of Biostatistics, Gillings School of Global Public Health, The University of North Carolina at Chapel Hill,; 10 Center for Health Promotion and Disease Prevention, The University of North Carolina at Chapel Hill

## Abstract

**Importance:**

Colorectal cancer (CRC) screening is effective but remains underused in federally qualified health centers (FQHCs).

**Objective:**

To assess the effectiveness of a centralized CRC screening outreach intervention involving mailed fecal immunochemical testing (FIT) outreach and patient navigation to colonoscopy after abnormal results of FIT.

**Design, Setting, and Participants:**

A pragmatic randomized clinical trial was conducted, using intention-to-treat analysis. Participants were enrolled from July 6, 2020, to September 17, 2021, and analyses were performed from July 6, 2023, to January 31, 2024. The study was conducted at independent FQHCs comprising 12 clinical delivery sites in North Carolina. The outreach intervention was centralized at an academic cancer center. Active individuals aged 50 to 75 years at average risk for CRC and not current with screening per US Preventive Services Task Force recommendations were included.

**Intervention:**

In addition to usual care, intervention participants received mailed screening outreach materials including an introductory letter, FIT kit packet with instructions and return postage, and 2 reminder letters if needed. Intervention participants with positive results of mailed FIT were offered navigation to facilitate follow-up colonoscopy completion. Control participants received usual care alone.

**Main Outcomes and Measures:**

The primary outcome was completion of a US Preventive Services Task Force–recommended CRC screening test within 6 months determined by electronic health record review. Secondary outcomes were colonoscopy completion within 6 months after positive FIT results and detection of advanced colorectal neoplasia, defined as advanced adenoma or CRC.

**Results:**

A total of 4002 participants were included (mean [SD] age, 59.6 [6.8] years; 2256 [56.4%] female; 364 (9.1%) Hispanic; 1082 [27.0%] non-Hispanic Black; 2288 [57.2%] non-Hispanic White; 1198 [29.9%] commercially insured; 617 [15.4%] Medicaid; 1227 [30.7%] Medicare; and 960 [24.0%] uninsured), with 2001 randomized to each group. Compared with controls, intervention participants were more likely to complete screening within 6 months of randomization (30.0% vs 9.7%; difference, 20.29 percentage points; 95% CI, 17.85-22.73 percentage points). The intervention was effective in all insurance types. In the intervention arm, 33 of 48 participants with positive FIT results (68.8%) completed follow-up colonoscopy within 6 months compared with 8 of 18 participants (44.4%) in the control arm (difference, 24.3 percentage points; 95% CI, −2.13 to 50.74 percentage points). Advanced colorectal neoplasia was detected in 29 intervention participants (1.4%) and 15 control participants (0.7%) (difference, 0.68 percentage points; 95% CI, 0.05-1.35 percentage points).

**Conclusions and Relevance:**

In this randomized clinical trial of centralized screening outreach intervention in diverse patients served by independent FQHCs, CRC screening completion and advanced colorectal neoplasia detection were substantially increased. Future studies should examine the cost and scalability of this intervention in this context.

**Trial Registration:**

ClinicalTrials.gov Identifier: NCT04406714

## Key Points

**Question:**

Does adding centralized mailed fecal immunochemical testing and patient navigation to usual care improve colorectal cancer (CRC) screening in US federally qualified health centers?

**Findings:**

This randomized clinical trial in 4002 individuals who were not current with CRC screening found that 30% of those who received the intervention completed screening within 6 months compared with 10% of those who received usual visit-based care alone. This was a statistically significant finding.

**Meaning:**

The findings of this study suggest that centralized mailed fecal immunochemical testing outreach can substantially improve CRC screening in diverse patient populations served in federally qualified health centers.

## Teaser

This randomized clinical trial examines the effect of an outreach intervention with mailed fecal immunochemical testing kits on receipt of colorectal cancer (CRC) screening among adults with average risk for CRC.

## Introduction

Despite compelling evidence that colorectal cancer (CRC) screening reduces CRC mortality, screening is severely underused in federally qualified health centers (FQHCs), which serve more than 30 million low-income individuals in the US.^1,2^ Accumulating evidence supports intervention strategies including organized mailed fecal immunochemical test (FIT) outreach directly to individuals to improve screening.^3^

Sustained implementation of organized mailed FIT has historically been confined to large, integrated health care delivery systems serving insured populations, often with a single insurance coverage type. Examples include the Veterans Health Administration or Kaiser Permanente.^4,5^ In contrast to these integrated health care delivery settings, mailed FIT has seen limited uptake in nonintegrated safety-net primary care contexts, such as FQHCs, which are constrained by care fragmentation and limited resources, making implementation of organized screening outreach challenging.^6^ Federally qualified health centers are operationally, legally, and fiscally independent from one another and from endoscopy services, which are essential for follow-up of abnormal stool test results. Each FQHC generally uses its own electronic health record (EHR) system. Federally qualified health centers serve historically marginalized communities with a varied payer mix and substantial numbers of uninsured or underinsured patients.^7^ These challenges are magnified in rural settings, where disparities in CRC screening and access to preventive services such as colonoscopy are acute.^8^

We developed a multilevel screening intervention centralized at an academic cancer center that conducted organized mailed FIT outreach and patient navigation to colonoscopy for patients with abnormal results of FITs. The intervention was developed in partnership with FQHCs serving diverse communities in North Carolina.^9^ Following a development phase, we conducted a pragmatic randomized clinical trial to assess the intervention’s effectiveness in 2 FQHC systems. Herein, we report the trial’s primary effectiveness outcome of CRC screening completion and secondary trial outcomes of colonoscopy completion after an abnormal stool test result and advanced colorectal neoplasia detection.

## Methods

### Study Context and Design

This randomized clinical trial was conducted as part of the National Cancer Institute Accelerating Colorectal Cancer Screening and Follow-Up Through Implementation Science research initiative.^10^ Details of the Scaling Colorectal Cancer Screening through Outreach, Referral, and Engagement (SCORE) trial, including design, setting, sampling protocol, and outcomes have been previously published.^11^ The trial protocol is available in Supplement 1. We followed the Consolidated Standards of Reporting Trials (CONSORT) reporting guideline for randomized clinical trials.^12^ Briefly, the randomized clinical trial tested the effectiveness of a multilevel intervention involving mailed FIT outreach and patient navigation to colonoscopy after abnormal FIT results compared with usual care alone on CRC screening in FQHCs. We enrolled active patients due for screening at 2 FQHCs comprising 12 primary care clinical delivery sites serving predominantly rural catchments in North Carolina. The 2 FQHCs were operationally independent, located in different geographic regions, served distinct populations, and had different EHR systems and endoscopy referral networks. Usual care consisted primarily of opportunistic, visit-based CRC screening, including FIT ordering or colonoscopy referral. The study was approved by the institutional review board at The University of North Carolina at Chapel Hill, with informed consent waived because this pragmatic trial assessed clinical effectiveness of this intervention. Individual informed consent would be infeasible and would introduce volunteer bias.^13^

### Participant Enrollment

We enrolled patients aged 50 to 75 years with at least 1 primary care visit in the past 18 months with average risk for CRC and not current with CRC screening per the US Preventive Services Task Force guidelines at the time of enrollment.^14^ We excluded individuals with inflammatory bowel disease, personal or family history of CRC, and major comorbidities as described in the protocol.^11^ To mimic clinical practice care and mitigate volunteer and selection bias, the trial was pragmatic. This was a pragmatic trial in that it aimed to mitigate volunteer and selection bias, mimic clinical practice, and increase applicability by eliminating the requirement for individual patient-level consent. Besides usual care, intervention patients received mailed FIT outreach materials that were branded to their FQHC, including site-specific letterhead and logos. Control arm patients received usual care and were not made aware that a study was being conducted.

### Study Activities and Randomization

Enrolled patients were randomized to intervention or control arms between July 6, 2020, and September 17, 2021, and assessed for CRC screening completion 6 months after randomization. Mailed outreach occurred over 15 waves, ranging from 100 to 400 participants randomized per wave. We determined eligibility using EHR data queries at the time of each wave. Because we sought to have sufficient power to obtain effectiveness estimates according to payer type, we aimed to draw equal samples from 4 main insurance strata (commercial, Medicaid, Medicare, and uninsured). If there were insufficient numbers of eligible individuals in one insurance category at the time of a given enrollment wave (eg, because a site had a small Medicaid population), we enrolled participants from the remaining categories to achieve the full final sample size. The biostatistician (X.T.) electronically generated the allocation sequence and one of us (A.T.B.) enrolled and assigned participants to interventions. Eligible patients were randomized in a 1:1 ratio using permuted block randomization with varying block sizes in each wave to receive either the intervention or control. Randomization was stratified by insurance category and FQHC site.

### Intervention 

The SCORE intervention is described in detail in the protocol and elsewhere.^9,11,15^ Briefly, the intervention consisted of an introductory (priming) letter followed 1 week later by a FIT kit packet that included educational and instructional inserts and a prepaid envelope to mail the stool sample to the laboratory. Participants who had not returned a completed test were sent up to 2 mailed reminders at approximately 2-week intervals. Navigators had immediate access to mailed FIT results, but not to FITs delivered in usual care, through the commercial laboratory. They offered telephone-based navigation to facilitate follow-up colonoscopy.^16^ Intervention components were provided in English and Spanish.

### Outcome Measures and Power Calculation

The primary outcome was completion of a US Preventive Services Task Force–recommended CRC screening test within 6 months of randomization. This was assessed through manual EHR review conducted independently by 2 research team members (C.M.R., L.R.S., and V.T.), who were blind to study arm assignment (dual independent review).^11^ We also determined the type of CRC screening test completed (ie, stool test or endoscopic test). Discrepancies among reviewers were resolved by consensus discussion involving a third blinded clinician investigator (D.S.R.).

Prespecified secondary outcomes included completion of colonoscopy within 6 months of an abnormal stool test result and detection of advanced colorectal neoplasia (including both advanced adenomas and CRC) within 12 months of randomization. Endoscopic findings were assessed by abstracting endoscopy and pathology reports for trial participants completing a colonoscopy during the 12 months following randomization. Abstractors (C.M.R., L.R.S., and C.S.) were blind to study arm assignment, and findings were categorized as (1) normal endoscopy, (2) low-risk adenoma, (3) 3 or more adenomas, and (4) advanced colorectal neoplasia (ie, advanced adenoma or CRC), based on 2020 US Multi-Society Task Force on Colorectal Cancer criteria.^17^ Two of us (D.S.R. and S.D.C.) who were blind to study arm assignment reviewed and confirmed CRC findings and adjudicated categorization when the primary abstractors were uncertain. We calculated that 3936 total participants (492 per arm per insurance strata) would provide 80% power to detect an 8 percentage-point difference in CRC screening completion between study arms.^11^

### Statistical Analysis

We conducted intention-to-treat analysis to assess the difference in the proportion of participants meeting the primary outcome (ie, completion of a US Preventive Services Task Force–recommended CRC screening test within 6 months) using the Cochran-Mantel-Haenszel test, controlling for insurance type and clinical site. The Breslow-Day test was used to check whether this association varied across insurance categories. We generated 2-sided 95% Newcombe CIs for the difference in proportions for the overall trial cohort and for each insurance category in preplanned, insurance-stratified analyses. We used Bonferroni adjustment for multiple tests.^11^ For secondary outcomes, we calculated colonoscopy completion rates within 6 months of an abnormal stool test result in each study arm and compared these rates between study arms using χ^2^ tests. Detection rates of advanced colorectal neoplasia at 12 months were also compared across study arms using χ^2^ tests. Analyses were conducted from July 6, 2023, to January 31, 2024, using R, version 4.3.3 (R Foundation for Statistical Computing) and SAS, version 9.4 (SAS Institute Inc).

## Results

**Figure 1.**
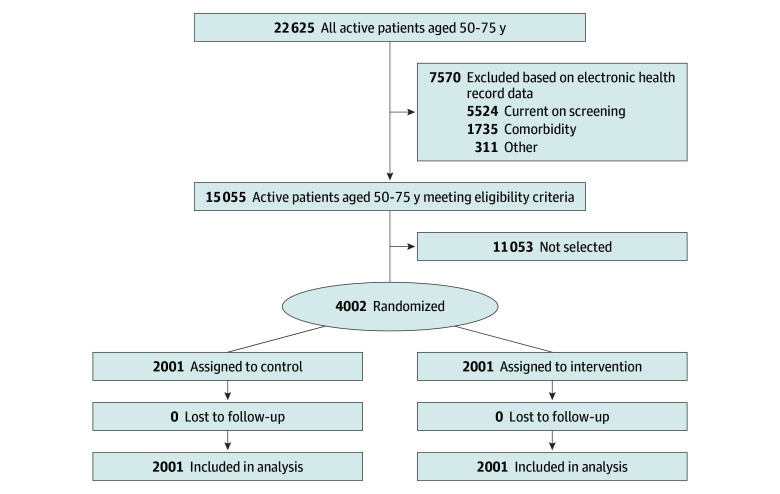
Patient Flowchart Twenty-eight patients (14 intervention, 14 control) were found retrospectively to have completed screening in the 30 days before randomization; all were included in the analysis.

Figure 1 shows participant study flow. Using queries of the 2 FQHC EHR systems, we identified a total of 15 055 patients aged 50 to 75 years without evidence of current CRC screening or other exclusion criteria during the study period. Of these, 4002 were sampled in waves based on our enrollment scheme and randomized, with 2001 participants in each arm.

Participant characteristics (Table 1) based on EHR data included mean (SD) age, 59.6 (6.8) years; 2256 (56.4%) female; 1746 (43.6%) male; 364 (9.1%) Hispanic; 1082 (27.0%) non-Hispanic Black; 2288 (57.2%) non-Hispanic White; 271 (6.8%) Spanish language preference; 1198 (29.9%) commercially insured; 617 (15.4%) Medicaid; 1227 (30.7%) Medicare; and 960 (24.0%) uninsured. Most individuals (3026 [75.6%]) had no prior evidence of CRC screening per EHR records.

**Table 1.** Characteristics of Trial Participants

Characteristic^a^	Participants, No. (%)		
	Control (n = 2001)	Intervention (n = 2001)	Total (N = 4002)
Age, mean (SD), y	59.6 (6.8)	59.7 (6.7)	59.6 (6.8)
Age category, y			
50-54	587 (29.3)	561 (28.0)	1148 (28.7)
55-59	467 (23.3)	477 (23.8)	944 (23.6)
60-64	442 (22.1)	449 (22.4)	891 (22.3)
65-69	280 (14.0)	323 (16.1)	603 (15.1)
70-75	225 (11.2)	191 (9.6)	416 (10.4)
Sex			
Female	1121 (56.0)	1135 (56.7)	2256 (56.4)
Male	880 (44.0)	866 (43.3)	1746 (43.6)
Race and ethnicity^b^			
Hispanic	179 (8.9)	185 (9.2)	364 (9.1)
Non-Hispanic Black	533 (26.6)	549 (27.4)	1082 (27.0)
Non-Hispanic White	1157 (57.8)	1131 (56.5)	2288 (57.2)
Other/unknown	132 (6.2)	136 (6.8)	268 (6.7)
Preferred language			
English	1462 (73.1)	1456 (72.8)	2918 (72.9)
Spanish	136 (6.8)	135 (6.7)	271 (6.8)
Other/unknown^c^	404 (20.2)	410 (20.5)	812 (20.3)
Primary insurance			
Commercial	598 (29.9)	600 (30.0)	1198 (29.9)
Medicaid	308 (15.4)	309 (15.4)	617 (15.4)
Medicare	614 (30.7)	613 (30.6)	1227 (30.7)
Uninsured	481 (24.0)	479 (23.9)	960 (24.0)
Prior CRC screening per EHR records^d^			
Stool testing	454 (22.7)	467 (23.3)	921 (23.0)
Endoscopy	31 (1.5)	25 (1.2)	55 (1.4)
None	1517 (75.8)	1509 (75.4)	3026 (75.6)

Abbreviations: CRC, colorectal cancer; EHR, electronic health record.

^a^ Source was the EHR data at the participating federally qualified health center sites.

^b^ Combined US census categories for race and ethnicity. In the other/unknown category, race information was available for 44 individuals in the EHR (11 American Indian or Alaska Native, 27 Asian, 3 Native Hawaiian/Pacific Islander, and 3 some other race), and missing for 224 individuals.

^c^ Other/unknown category includes 15 individuals who reported a language other than English or Spanish (Chinese, French, Hindi, North American Indian, Norwegian, Oromo, Polish, Russian, Tagalog, and Vietnamese); 797 individuals did not have a preferred language listed in the EHR.

^d^ Patients who had previously completed both a stool test and an endoscopic test are classified in endoscopy category. Stool testing included fecal immunochemical testing DNA and guaiac fecal occult blood test.

Participant characteristics varied substantially by site, reflecting different populations served by these FQHCs (eTable in Supplement 2). Most notably, patients at FQHC site 1 (n = 2000) were more likely to be Hispanic (15.6% vs 2.7%) or non-Hispanic White (73.1% vs 41.3%), Medicaid enrollees (22.1% vs 8.7%), and to have previously completed a stool test for CRC screening per EHR records (28.5% vs 17.5%). Patients at FQHC site 2 (n = 2002) were more likely than those at site 1 to be non-Hispanic Black (49.4% vs 4.7%) and have Medicare (35.4% vs 26.0%).

### Primary Outcome

Table 2 reports the results for the primary outcome of CRC screening completion. Overall, compared with control participants, those assigned to the intervention arm were more likely to complete screening within 6 months of randomization: 30.0% vs 9.7% (difference, 20.29 percentage points; 95% CI, 17.85-22.73 percentage points). Colorectal cancer screening completion rates at 12 months were 34.6% vs 16.6% (difference, 17.99 percentage points; 95% CI, 15.30-20.69 percentage points). Of the 600 intervention patients who completed CRC screening at 6 months, 519 (86.5%) completed a stool test alone and 81 (13.5%) completed a colonoscopy (either after abnormal FIT result or without FIT screening). Of the 194 patients in the control arm who completed screening, 127 (65.5%) completed a stool test alone and 67 (34.5%) completed a colonoscopy.

**Table 2.** Difference in Proportion of CRC Screening Completion Within 6 Months by Arm

Subgroup	No. (%)		Completed screening, No. (%)		Difference, percentage points (95% CI)^a^^,^^b^
	Intervention	Control	Intervention	Control	
All	2001 (100)	2001 (100)	600 (30.0)	194 (9.7)	20.29 (17.85-22.73)
Insurance type					
Commercial	600 (30.0)	598 (29.9)	186 (31.0)	54 (9.0)	21.97 (17.45-26.49)
Medicaid	309 (15.4)	308 (15.4)	91 (29.4)	28 (9.1)	20.36 (14.02-26.69)
Medicare	613 (30.6)	614 (30.7)	206 (33.6)	71 (11.6)	22.04 (17.36-26.72)
Uninsured	479 (23.9)	481 (24.0)	117 (24.4)	41 (8.5)	15.90 (11.11-20.70)

Abbreviation: CRC, colorectal cancer.

^a^ Two-sided 95% Newcombe CIs for the overall cohort and insurance-stratified analysis.

^b^ *P* values for the overall cohort were based on the Cochran-Mantel-Haenszel (CMH) test controlling for insurance and site. The *P* values for the insurance-specific analyses are based on the CMH test controlling for site; all differences were significant at *P* < .001. Differences remained statistically significant after Bonferroni correction.

Table 2 also presents primary outcome findings with stratified analysis by insurance type. Point estimates for intervention effects for insurance types ranged from approximately 22.00 percentage points (commercial, 21.97; Medicare, 22.04) to 15.90 percentage points (uninsured). However, the 95% CIs for all insurance types overlapped, and the Breslow-Day test showed no significant heterogeneity of odds ratios across strata (χ^2^ = 5.46; *P* = .60), suggesting no statistically significant differences in effectiveness by insurance type and site.

### Abnormal FIT and Colonoscopy Follow-Up 

Fecal immunochemical testing results were positive in 48 of 554 patients (8.7%) in the intervention arm and 18 of 134 patients (13.4%) in the control arm. In the intervention arm, 33 of 48 participants (68.8%) with abnormal FIT results completed a follow-up colonoscopy within 6 months of the test result. In the control arm, 8 of 18 patients (44.4%) with abnormal FIT results completed a follow-up colonoscopy within 6 months (difference, 24.3 percentage points; 95% CI, −2.13 to 50.74 percentage points; *P* = .07).

### Endoscopic Findings

**Figure 2.**
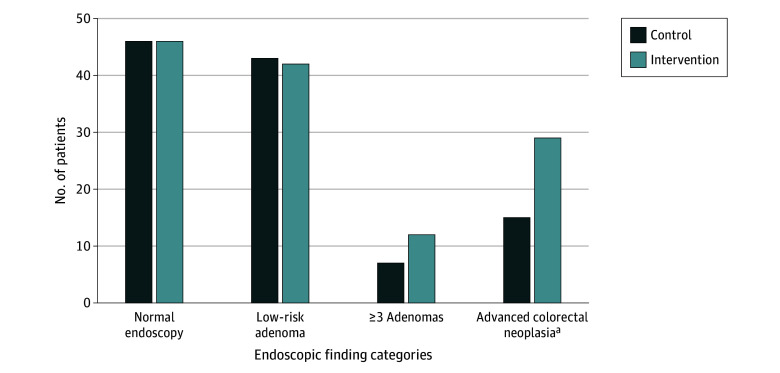
Endoscopic Outcomes Analysis in patients who completed at least 1 endoscopic test up to 12 months after randomization (control: 111; intervention: 129). Outcomes were not determined in 8 patients due to missing endoscopic reports. ^a^Advanced colorectal neoplasia includes advanced adenomas and colorectal cancer diagnoses (n = 7). Most patients diagnosed with colorectal cancer were in the intervention arm (n = 5).

We obtained endoscopy and pathology outcomes data on 240 of 248 participants (96.8%) who underwent colonoscopy within 12 months following randomization, permitting Multi-Society Task Force on Colorectal Cancer classification (Figure 2). Among intervention participants, 29 (1.4%) were found to have advanced colorectal neoplasia compared with 15 participants in the control arm (0.7%) (difference, 0.68 percentage points; 95% CI, 0.05-1.35 percentage points; *P* = .03).

### Deaths

We observed 42 deaths in this study cohort within 12 months after randomization (25 intervention, 17 control). There were 3 CRC-associated deaths (1 intervention, 2 control).

## Discussion

This centralized intervention involving mailed FIT and navigation for abnormal FIT results improved CRC screening in FQHCs, yielding a substantial increase in screening completion compared with usual care alone. The intervention was effective across payer types, including Medicaid and uninsured. This pragmatic study suggests that this kind of centralized intervention can be effective in addressing persistent cancer screening challenges experienced by independent FQHCs operating in a relatively fragmented care delivery environment.^2^

Our findings can be contextualized through comparison with the STOP CRC trial, a cluster randomized trial of mailed FIT outreach conducted in a similar context of FQHC clinics.^18^ In that study, tools (including outreach materials, EHR tools, training, and practice facilitation) were provided to intervention clinics, and on-site clinic staff at each clinic were responsible for implementing the intervention. STOP CRC found a 3.8 percentage–point increase in screening over usual care (18.3% vs 14.5%). The modest effect was likely due to wide clinic-level variation in implementation fidelity. Implementation challenges were explored qualitatively and attributed to clinic staff time burden, EHR staffing issues, and leadership turnover at intervention clinics.^19^ By contrast, the SCORE trial tested a different implementation approach of centralizing the outreach intervention at our state cancer center.^10^ The centralized team provided assistance with building and testing EHR tools and queries, conducting mailings, and navigating patients with abnormal FIT results to colonoscopy. This essentially permitted FQHC clinics to outsource most of their CRC screening outreach activities. This approach led to a 20.3 percentage–point increase (30.0% vs 9.7%) in screening completion compared with usual care.

Our findings complement those of other studies describing the benefits of mailed FIT outreach in increasing CRC screening in less-fragmented care contexts.^3,20,21,22^ For example, mailed FIT outreach has also been found to increase screening in populations having a specific payer, such as Medicaid or Medicare beneficiaries,^23,24,25,26^ in highly integrated health care systems, such as the Veterans Health Administration or Kaiser Permanente,^4,25^ and in large, integrated, and publicly funded urban safety-net settings.^27,28^

Our study also adds to literature illustrating important tradeoffs between organized screening outreach programs aimed at insurance beneficiary populations (defined by having a specific payer) vs patient populations (defined by receiving primary care at a particular health center or system).^15^ On the one hand, interventions such as ours delivered at the FQHC or health system level can improve screening across all eligible patient populations regardless of insurance status or type. On the other hand, payer-based interventions have the advantage of ensuring there is financial access to colonoscopy after an abnormal FIT result. Furthermore, payers can often dedicate staff resources that are not subject to challenges of competing clinical demands and staff turnover seen in FQHC settings.^6^ Moreover, payers may theoretically have a vested financial interest in reducing future need to cover costly treatment of advanced CRC disease. Nevertheless, the fact that patient populations change insurance plans frequently diminishes this incentive.^29,30,31^

Our intervention also included navigation of patients to colonoscopy for abnormal FIT results. A description of the navigation component of the intervention, including types of barriers addressed during navigation, is published elsewhere.^16^ Although navigation of patients with abnormal FIT results did not affect the primary outcome, which only required completion of any recommended CRC screening test, timely colonoscopy completion was an important secondary outcome because it is essential for effective screening.^32^ We found that 68.8% of patients with an abnormal FIT result in the intervention arm completed follow-up colonoscopy within 6 months, compared with 44.4% in the control arm. Although this difference was not statistically significant (*P* = .07), our study was not powered for this secondary outcome.

We observed a higher rate of FIT positivity in the control vs intervention arms (13.4% vs 8.7%), a finding attributable to a higher FIT positivity rate in the FIT brand (InSure; InSure One) used in usual care at one of the FQHC sites.^33^ The relatively high overall FIT positivity rate we observed may also be partly attributable to the fact that this is a highly unscreened population. A high FIT positive rate was also observed in the STOP CRC trial (13.6%) screening study conducted in a similar context.^18^

Our trial identified more individuals with advanced colorectal neoplasia (a prespecified outcome) in the intervention arm than in the control arm—a statistically significant finding. This lends support to the idea that mailed FIT interventions, particularly when coupled with navigation to follow-up colonoscopy after abnormal FIT results, offer an efficient way to use endoscopic resources, which are especially limited in rural parts of the US, to identify clinically important colorectal lesions.^34,35^

### Strengths and Limitations

A strength of this trial is the pragmatic design. Another strength of this study was sampling and stratification by insurance type, allowing us to examine effectiveness by payer type. We are currently conducting cost and cost-effectiveness analyses based on these data and findings of other economic assessments, such as time-and-motion observations and constituent-engaged system mapping exercises, to understand intervention implementation costs.^36,37^ These will inform future engagement with payers and regional partners around scaling and sustainability.

This study also has limitations. First, because it relied on EHR data, some individuals were retrospectively found to have been current with CRC screening after randomization. Also, some individuals could have moved and/or obtained CRC screening outside the area. However, our methods for obtaining and classifying relevant data using dual independent review reduced the risk of misclassification and missingness, and the randomized design makes differential misclassification of eligibility or outcomes across study arms highly unlikely. Second, this study examined the effect of a single round of mailed FIT. Effectiveness of repeated rounds needs to be established. Third, due to randomization at the patient level, contamination of usual care through increased clinician attention to CRC screening is possible. However, if it occurred, such contamination would increase usual care screening and reduce the observed intervention effect size (ie, bias toward the null effect). Fourth, this trial occurred during the COVID-19 pandemic, a period when clinical care processes were frequently disrupted and/or subject to rapid change. This may have affected our results by reducing screening completion in usual care and could affect the generalizability of our findings. However, we note that in trials conducted in similar populations before the pandemic, the observed screening completion rates in usual care are similar. Our trial found 16.6% completed screening in the control arm at 12 months, compared with 14.5% for the STOP CRC trial^18^ and 12.1% in the Gupta et al^28^ study. This suggests that, even without pandemic disruptions, the rates of usual care screening completion in largely previously unscreened populations in safety-net FQHCs tend to be low.

## Conclusions

In this pragmatic randomized clinical trial, we found that a centralized mailed FIT outreach intervention and patient navigation effectively increased CRC screening among diverse populations served by independent FQHCs. The intervention was effective across all insurance categories and was associated with increased detection of advanced colorectal neoplasia. Future work will examine cost issues that can inform policy decision-making regarding spread and sustainment with payers and other entities that may be able to provide similar types of centralized support, such as state community health center associations or cancer centers.

## Notes

Supplement 1.
Trial Protocol

Supplement 2. **eTable.** Characteristics of Participants by Site

Supplement 3.
Data Sharing Statement
