# Supplementary material for: Centralized Colorectal Cancer Screening Outreach in Federally Qualified Health Centers: A Randomized Clinical Trial
Source: JAMA Netw Open. 2024 Nov 25;7(11):e2446693. doi: 10.1001/jamanetworkopen.2024.46693 (PMC11589799; doi:10.1001/jamanetworkopen.2024.46693)
Supplement: Supplement 2. — eTable. Characteristics of Participants by Site [file jamanetwopen-e2446693-s002.pdf]

Supplementary Online Content

Reuland DS, O'Leary MC, Crockett SD, et al. Centralized colorectal cancer screening outreach in federally qualified health centers: a randomized clinical trial. *JAMA Netw Open*. 2024;7(11):e2446693. doi:10.1001/jamanetworkopen.2024.4669

eTable. Characteristics of Participants by Site

This supplementary material has been provided by the authors to give readers additional information about their work.

© 2024 Reuland et al. *JAMA Network Open.*

eTable. Characteristics of Participants by Site

**Characteristic**[a]	**Site 1 (N=2,000)**	**Site 2 (N=2,002)**	**All (N=4,002)**
Age, mean (SD)	59.4 (6.6)	59.8 (6.9)	59.6 (6.8)
Age category			
50-54	594 (29.7)	554 (27.7)	1,148 (28.7)
55-59	487 (24.4)	457 (22.8)	944 (23.6)
60-64	432 (21.6)	459 (22.9)	891 (22.3)
65-69	304 (15.2)	299 (14.9)	603 (15.1)
70-75	183 (9.2)	233 (11.6)	416 (10.4)
Sex			
Female	1,152 (57.6)	1,104 (55.1)	2,256 (56.4)
Male	848 (42.4)	898 (44.9)	1,746 (43.6)
Race/Ethnicity[b]			
Hispanic	311 (15.6)	53 (2.7)	364 (9.1)
Non-Hispanic Black	93 (4.7)	989 (49.4)	1,082 (27.0)
Non-Hispanic White	1,462 (73.1)	826 (41.3)	2,288 (57.2)
Other/Unknown	134 (6.7)	134 (6.7)	268 (6.7)
Preferred language[c]			
English	1,651 (82.6)	1,267 (63.3)	2,918 (72.9)
Spanish	242 (12.1)	29 (1.5)	271 (6.8)
Other/Unknown	108 (5.4)	706 (35.2)	812 (20.3)
Primary insurance[d]			
Commercial	520 (26.0)	678 (33.9)	1,198 (29.9)
Medicaid	443 (22.1)	174 (8.7)	617 (15.4)
Medicare	519 (26.0)	708 (35.4)	1,227 (30.7)
Uninsured	518 (25.9)	442 (22.1)	960 (24.0)
Prior CRC screening per EHR records[e]			
Yes, stool testing	570 (28.5)	351 (17.5)	921 (23.0)
Yes, endoscopy	55 (2.8)	0 (0)	55 (1.4)
No	1,375 (68.8)	1,651 (82.5)	3,026 (75.6)

[a] Source: electronic health record (EHR) data at the participating CHC sites.

[b] Combined US Census categories for race/ethnicity. In the Other/Unknown category, race information was available for 44 individuals in the EHR (11 American Indian/Alaskan Native, 27 Asian, 3 Native Hawaiian/Pacific Islander, and 3 some other race), and not available for 224 individuals.

[c] Other/Unknown category includes 15 individuals who reported a language other than English or Spanish (specifically Chinese, French, Hindi, North American Indian, Norwegian, Oromo, Polish, Russian, Tagalog, and Vietnamese); 797 individuals did not have a preferred language listed in the EHR.

© 2024 Reuland et al. *JAMA Network Open.*

[d] We attempted to sample patients from all four insurance status groups evenly. In cases where there were too few patients in a particular group at one of the sites, we enrolled patients from other insurance categories.

[e] Patients who had previously completed both a stool test and an endoscopic test are classified in endoscopy category. Stool testing included FIT DNA and guaiac FOBT.

© 2024 Reuland et al. *JAMA Network Open.*
