# Supplementary material for: Centralized Colorectal Cancer Screening Outreach in Federally Qualified Health Centers: A Randomized Clinical Trial
Source: JAMA Netw Open. 2024 Nov 25;7(11):e2446693. doi: 10.1001/jamanetworkopen.2024.46693 (PMC11589799; doi:10.1001/jamanetworkopen.2024.46693)
Supplement: Supplement 3. — Data Sharing Statement [file jamanetwopen-e2446693-s003.pdf]

Data Sharing Statement

Reuland. Centralized Colorectal Cancer Screening Outreach in Federally Qualified Health Centers. *JAMA Netw Open*. Published November 25, 2024.
doi:10.1001/jamanetworkopen.2024.46693

Data
Additional Information: The trial was registered at ClinicalTrials.gov (identifier NCT04406714).
Data available: Yes
Data types: Deidentified participant data
How to access data: https://healthcaredelivery.cancer.gov/accsis/datashare
When available: With publication

Supporting Documents
Document types: Other (please specify)
Additional Information: Codebook
How to access documents: https://healthcaredelivery.cancer.gov/accsis/datashare
When available: With publication

Additional Information
Who can access the data: Anyone requesting the data.
Types of analyses: For any purpose.
Mechanisms of data availability: By submitting a request through the ACCSIS DataShare website.
